# Na_v_1.7 expression is increased in painful human dental pulp

**DOI:** 10.1186/1744-8069-4-16

**Published:** 2008-04-21

**Authors:** Songjiang Luo, Griffin M Perry, S Rock Levinson, Michael A Henry

**Affiliations:** 1 Department of Endodontics, University of Texas Health Science Center at San Antonio, San Antonio, TX 78229, USA; 2 Department of Physiology and Biophysics, University of Colorado at Denver and Health Sciences Center, Aurora, CO 80045, USA

## Abstract

**Background:**

Animal studies and a few human studies have shown a change in sodium channel (NaCh) expression after inflammatory lesions, and this change is implicated in the generation of pain states. We are using the extracted human tooth as a model system to study peripheral pain mechanisms and here examine the expression of the Na_v_1.7 NaCh isoform in normal and painful samples. Pulpal sections were labeled with antibodies against: 1) Na_v_1.7, N52 and PGP9.5, and 2) Na_v_1.7, caspr (a paranodal protein used to identify nodes of Ranvier), and myelin basic protein (MBP), and a z-series of optically-sectioned images were obtained with the confocal microscope. Na_v_1.7-immunofluorescence was quantified in N52/PGP9.5-identified nerve fibers with NIH ImageJ software, while Na_v_1.7 expression in myelinated fibers at caspr-identified nodal sites was evaluated and further characterized as either typical or atypical as based on caspr-relationships.

**Results:**

Results show a significant increase in nerve area with Na_v_1.7 expression within coronal and radicular fiber bundles and increased expression at typical and atypical caspr-identified nodal sites in painful samples. Painful samples also showed an augmentation of Na_v_1.7 within localized areas that lacked MBP, including those associated with atypical caspr-identified sites, thus identifying NaCh remodeling within demyelinating axons as the basis for a possible pulpal pain mechanism.

**Conclusion:**

This study identifies the increased axonal expression and augmentation of Na_v_1.7 at intact and remodeling/demyelinating nodes within the painful human dental pulp where these changes may contribute to constant, increased evoked and spontaneous pain responses that characterize the pain associated with toothache.

## Background

The activation of voltage-gated sodium channels (NaChs) plays an essential role in neuronal excitability, including the initiation and propagation of action potentials [1]. Experimental animal studies and a few human studies have shown that NaChs change their expression in sensory neurons following inflammatory and nerve lesions and these changes may contribute to the activation of pain pathways leading to the development of increased pain states [2].

The NaChs represent a diverse gene family, with at least nine different isoforms identified within the mammalian nervous system [3]. These different isoforms not only show distinct electrophysiological properties but also select distributions within different regions of the nervous system [4]. Much interest has been placed on the contribution of isoforms preferentially expressed within the peripheral nervous system to pain states. Although there is evidence for the involvement of each of these isoforms in nociception, the Na_v_1.7 isoform has recently been most critically linked to pain in humans [4].

The human dental pulp is richly innervated by nociceptive primary afferents [5] and represents a common site of pathology and pain [6]. A common treatment modality includes the extraction of the painful tooth, whereas normal wisdom teeth are also routinely extracted. Moreover, pain characteristics can be documented prior to extraction, and together these features distinguish the use of the human dental pulp as a model system to evaluate peripheral pain mechanisms. Here, we use this model system to evaluate the expression of Na_v_1.7 within normal and painful human pulpal specimens with the use of quantitative methods and demonstrate increased axonal expression of Na_v_1.7 within axon bundles and at typical and atypical nodal sites that showed alterations in myelin staining relationships within painful samples.

## Results

### Qualitative description of Na_v_1.7 expression in N52/PGP9.5-identified nerves

**Figure 1:**
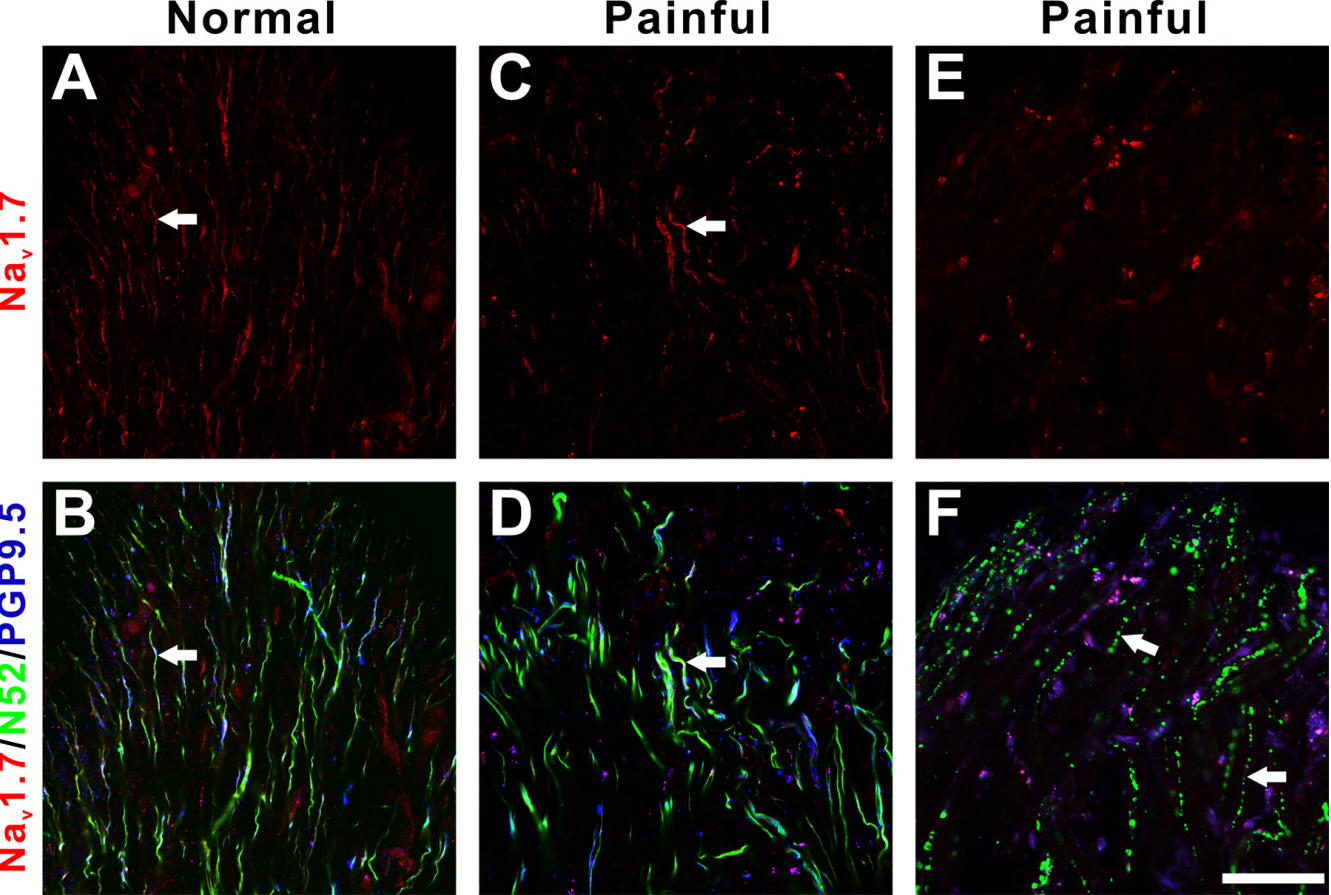
**Na_v_1.7 expression in pulp horn**. **A-F. **Confocal micrographs of single optical images showing Na_v_1.7 (red) expression within N52 (green) and PGP9.5 (blue)-identified nerve fibers within the pulp horn of one normal (A, B) and two painful (C-F) samples. **A **and **B. **Na_v_1.7 (arrow) is expressed in many nerve fibers in the normal pulp horn (A) that stain with N52 and PGP9.5 as seen in the combined image in B. **C **and **D. **The pulp horn from a painful sample also shows Na_v_1.7 expression (arrow) in most nerve fibers that stain with N52 and PGP9.5 as seen in the combined image in D. **E **and **F. **The pulp horn of one painful sample contains no Na_v_1.7 staining (E) within fibers that appear fragmented as visualized with N52 staining (F; arrows). Scale bars = 50 μm.

**Figure 2:**
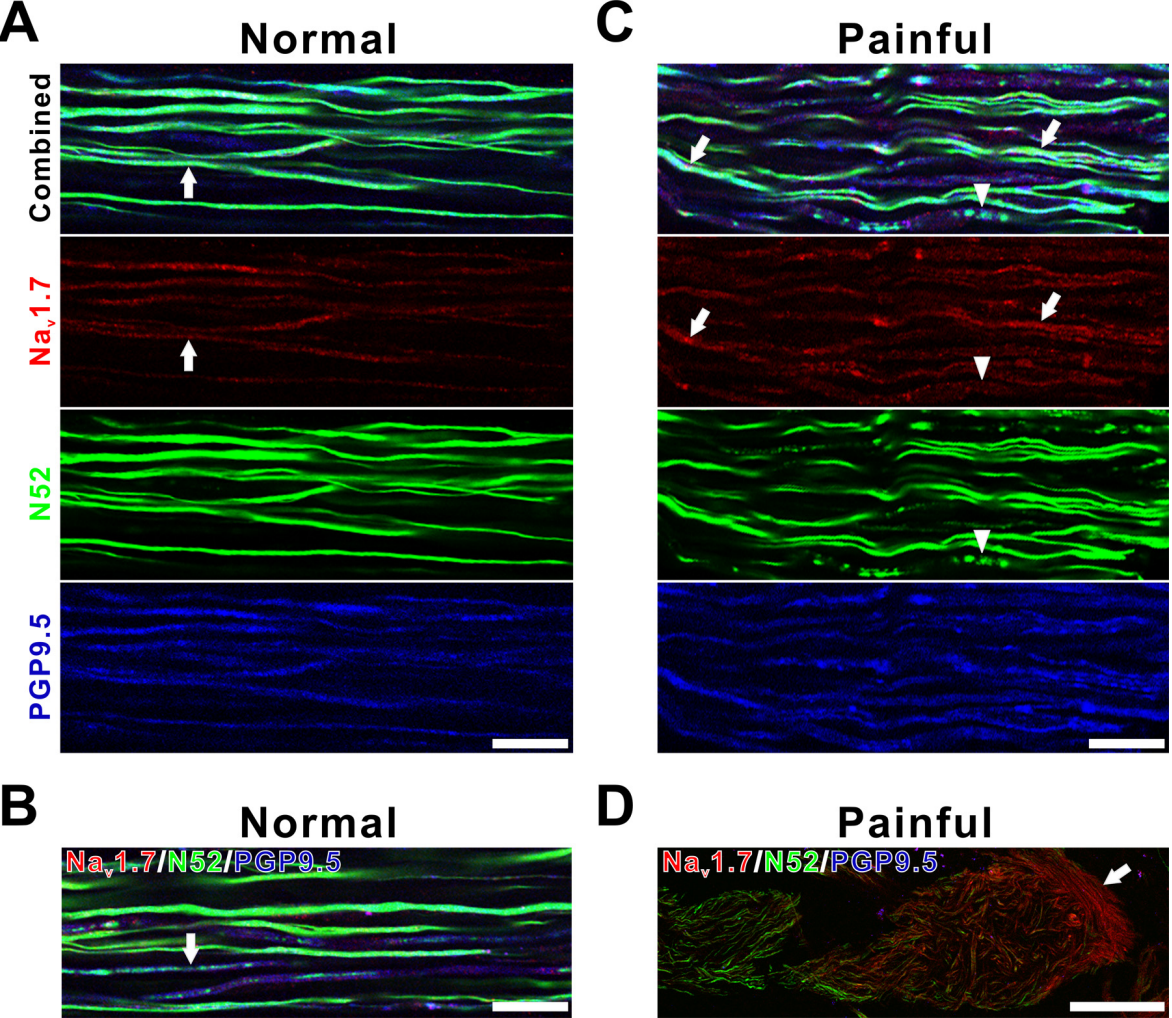
**Na_v_1.7 expression in axon bundles**. **A-D. **Confocal micrographs of single optical images showing Na_v_1.7 (red) expression within N52 (green) and PGP9.5 (blue)-identified nerve fibers within axon bundles in normal (A, B) and painful (C, D) samples. **A **and **B. **Na_v_1.7 (red; arrows) is expressed in nerve fibers that appear intact as visualized with PGP9.5 and especially N52 (green) staining within an axon bundle located in a normal sample (A), while a different normal sample shows some fibers that appear fragmented as seen with N52 staining (B; arrow). **C **and **D. **The expression of Na_v_1.7 within a painful sample (C) appears prominent within intact nerve fibers (arrows), but less in fibers that appear fragmented (arrowheads) as visualized with N52 staining. A different painful sample contains many fibers with prominent Na_v_1.7 staining, including some that show minimal staining with N52 and PGP9.5 (D; arrow). Scale bars = 20 μm in A-C and 100 μm in D.

Na_v_1.7-immunofluorescence was observed in many nerve fibers identified by N52/PGP9.5 staining in all regions of both normal and painful dental pulp samples, including fibers within the pulp horn (Fig. 1) and axon bundles located throughout the pulp (Fig. 2). Fibers with Na_v_1.7 staining most commonly included those with both N52 and PGP9.5 (Figs. 1A–D and 2A,C). The N52/PGP9.5 staining (and especially the N52 staining) was mostly seen within intact fibers in the normal samples (Fig. 2A), but some normal samples (Fig. 2B) and most painful samples (Fig. 2C) also contained isolated fibers that appeared fragmented. These fragmented fibers were generally intermixed among intact ones located within axon bundles. One painful sample contained only fragmented axons in the coronal pulp (Figs. 1E,F) and these appeared very different than those isolated fragmented axons seen in some normal (Fig. 2B) and most painful samples (Fig. 2C). The expression of Na_v_1.7 was minimal in fibers with a fragmented appearance (Figs. 1E,F and 2C), while the expression within fibers with an intact appearance was seen in different-sized fibers and included even staining of low intensity and focal accumulations with brighter intensity. Both of these staining patterns appeared more common in painful samples (compare Figs. 2A to 2C). Three of the painful samples contained localized areas within the middle of the coronal pulp with a high density of fibers with prominent Na_v_1.7 staining, but with a diminished intensity or even lack of N52/PGP9.5 staining within these same fibers (Fig. 2D). The Na_v_1.7 expression within fibers that lacked N52/PGP9.5 staining would not be evaluated with the quantitative analysis described below. Since increased numbers of accumulations with Na_v_1.7 were seen in painful samples, adjacent sections of four sample pairs were stained with caspr, a paranodal protein used to identify nodes of Ranvier [7] and myelin basic protein (MBP) antibodies for further characterization.

### Quantitative analysis of Na_v_1.7 expression in N52/PGP9.5-identified nerve area

**Figure 3:**
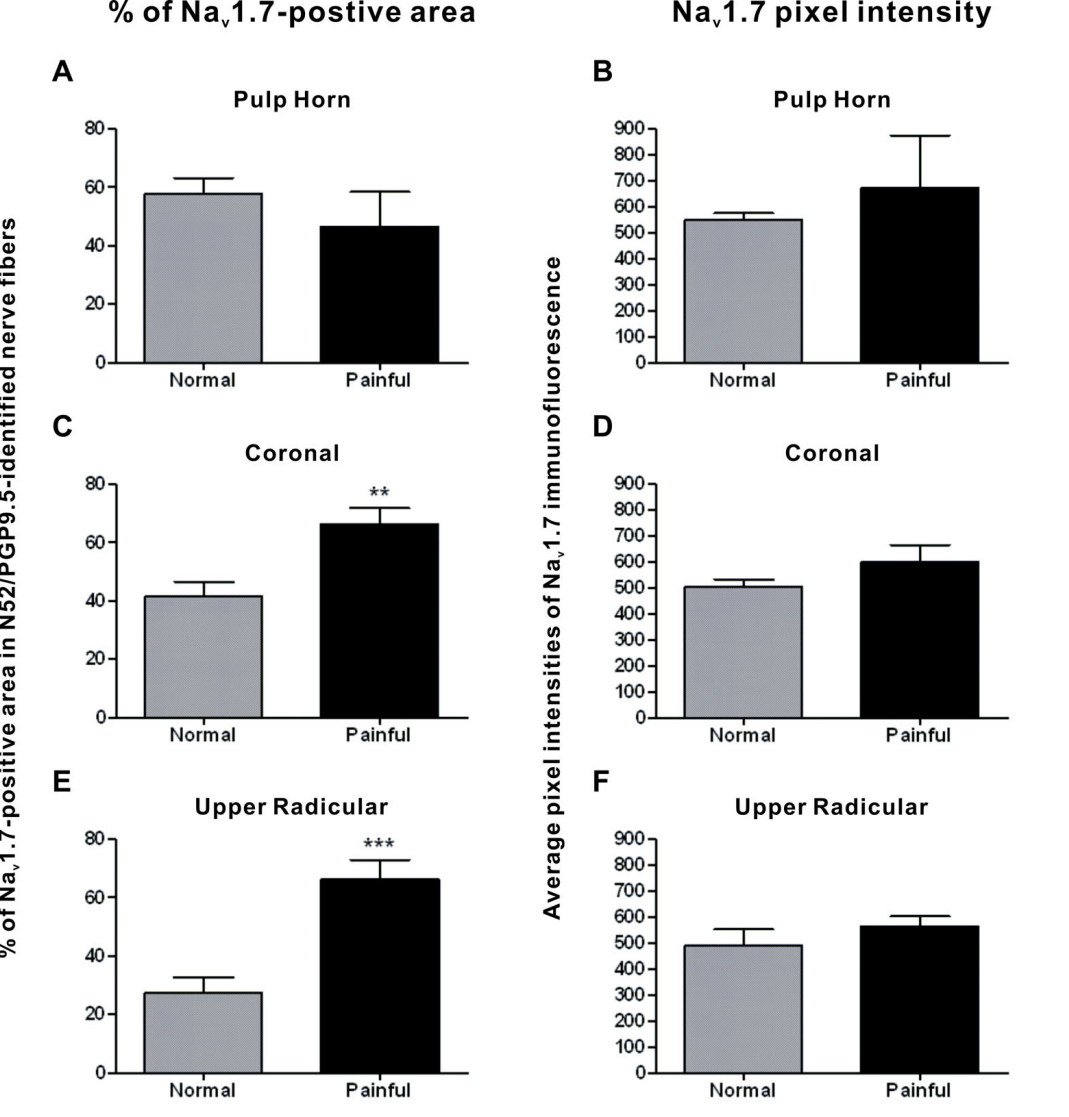
**Results of quantitative analyses of Na_v_1.7 expression within nerve fibers in normal and painful samples**. **A-F. **The nerve area and pixel intensity of Na_v_1.7 expression within N52/PGP9.5-identified nerve fibers was quantified within different regions in normal and painful samples. **A **and **B. **The nerve area (A) and pixel intensity (B) of Na_v_1.7 expression was not significantly different within the pulp horns of normal and painful samples. **C-F. **The nerve area of Na_v_1.7 expression was significantly greater within coronal (C) and upper radicular (E) axon bundles in painful samples, but with no difference between normal and painful samples in pixel intensity within these same regions (D, F). ** = p < 0.01, *** = p < 0.001.

Quantitative analysis showed no difference in the Na_v_1.7 expression within N52/PGP9.5-identified nerve area or in the pixel intensity of this expression within axons in the pulp horn of normal samples when compared to painful samples (area – 57.6% ± 5.6 vs. 46.6% ± 11.9; Fig. 3A/intensity – 549.6 ± 26.1 vs. 670.8 ± 204.7; Fig. 3B). In contrast, the nerve area occupied by Na_v_1.7-immunoreactivity was significantly greater within axon bundles located in the coronal (41.7% ± 4.8 vs. 66.5% ± 5.4; p < 0.01 – Fig. 3C) and the upper radicular (27.2% ± 5.5 vs. 66.0% ± 7.0; p < 0.001 – Fig. 3E) regions of the painful samples, but with no differences in pixel intensity in either of these areas (coronal – 503.8 ± 28.2 vs. 600.2 ± 65.4; Fig. 3D/upper radicular – 489.1± 63.4 vs. 564.8 ± 38.2; Fig. 3F).

### Na_v_1.7 expression and characterization at caspr-identified nodal sites

**Figure 4:**
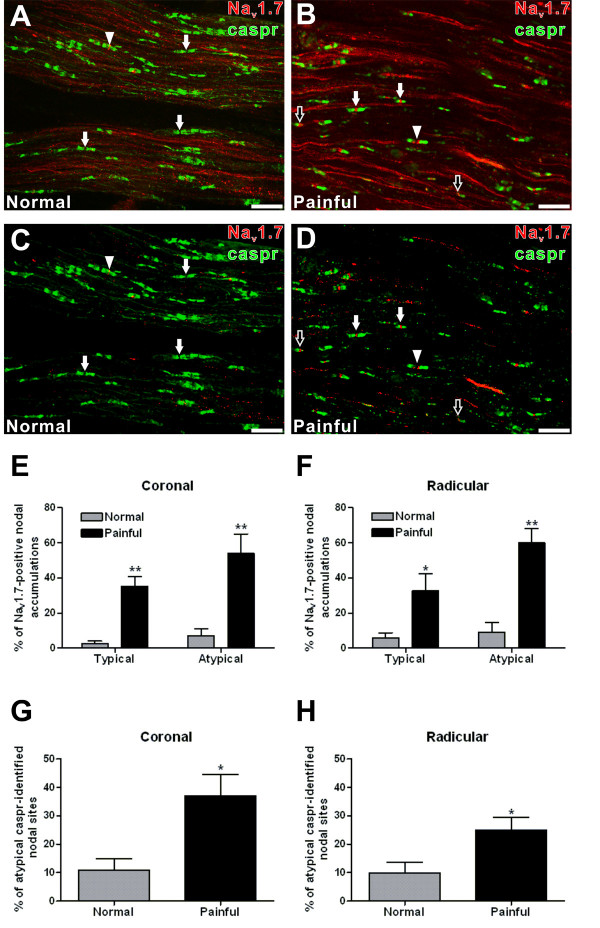
**Na_v_1.7 expression at caspr-identified nodal sites**. **A-D. **Confocal micrographs of maximum-intensity z-projections (five z-sections with spacing increments of one μm) showing the expression of non-thresholded (A, B) and thresholded (C, D) Na_v_1.7 (red) immunofluorescence at caspr (green)-identified sites within axon bundles in normal (A, C) and painful (B, D) samples. Typical nodal forms (arrows) predominate in the normal sample (A, C) and one of these shows Na_v_1.7 expression located within the nodal gap (arrowhead). The painful sample (B, D) shows atypical nodal forms that include both heminodes (black arrows) and a split node (arrowhead). Many of these atypical nodal forms and some of the typical nodal forms (white arrows) are associated with Na_v_1.7. **E **and **F. **The expression of Na_v_1.7 at typical and atypical caspr-identified nodal sites was determined within axon bundles located in coronal and radicular regions in normal and painful samples. Results show a significant increase in the percent of both typical and atypical nodes that were associated with Na_v_1.7 within the coronal (E) and radicular (F) axon bundles in painful samples. **G **and **H. **All caspr-identified nodal sites were evaluated within axon bundles located in coronal and radicular regions in normal and painful samples and were classified as either typical or atypical. Results show a significant increase in the percent of atypical nodal sites within the coronal (G) and radicular (H) axon bundles in painful samples. Scale bars = 20 μm; * = p < 0.05, ** p < 0.01.

Evaluation of pulpal tissue sections stained with caspr allowed a characterization of Na_v_1.7 at caspr-identified nodal sites that were classified as either typical or atypical as based on caspr relationships (see Methods for details). This analysis was limited to axon bundles within the coronal and upper radicular regions of four normal and four painful samples, since nodes are commonly seen in these regions. This analysis was performed on images that were thresholded to eliminate low intensity pixels while maintaining those with higher immunofluorescence intensity, since these are the ones typically seen at nodes of Ranvier (Figs. 4A–D). The total number of nodal sites evaluated was 2320 and included more than 500 sites in each region in both normal and painful samples. This analysis showed a dramatic increase in the percentage of nodal sites with Na_v_1.7 expression in painful samples within coronal and upper radicular regions (Figs. 4E,F). This increased percentage of Na_v_1.7-positive nodal accumulations within painful samples was seen at both typical (coronal – 2.7% ± 1.5 vs. 35.3% ± 5.5; p < 0.01 – Fig. 4E/upper radicular – 5.9% ± 2.7 vs. 32.7% ± 9.8; p < 0.05 – Fig. 4F) and atypical (coronal – 6.9% ± 4.0 vs. 53.9% ± 11.1; p < 0.01 – Fig. 4E/upper radicular – 9.1% ± 5.5 vs. 59.8% ± 8.4; p < 0.01 – Fig. 4F) nodal sites. In addition, there was a significant increase in the occurrence of atypical nodal forms within painful samples relative to the total number of all caspr-identified sites (coronal – 11.0% ± 3.9 vs. 36.9% ± 7.7; p < 0.05 – Fig. 4G/upper radicular – 9.9% ± 3.7 vs. 24.9% ± 4.6; p < 0.05 – Fig. 4H). Together, these results identify the increased association of Na_v_1.7 at both typical and atypical nodal sites and an increased occurrence of atypical nodal forms within painful samples.

### Myelin basic protein expression

**Figure 5:**
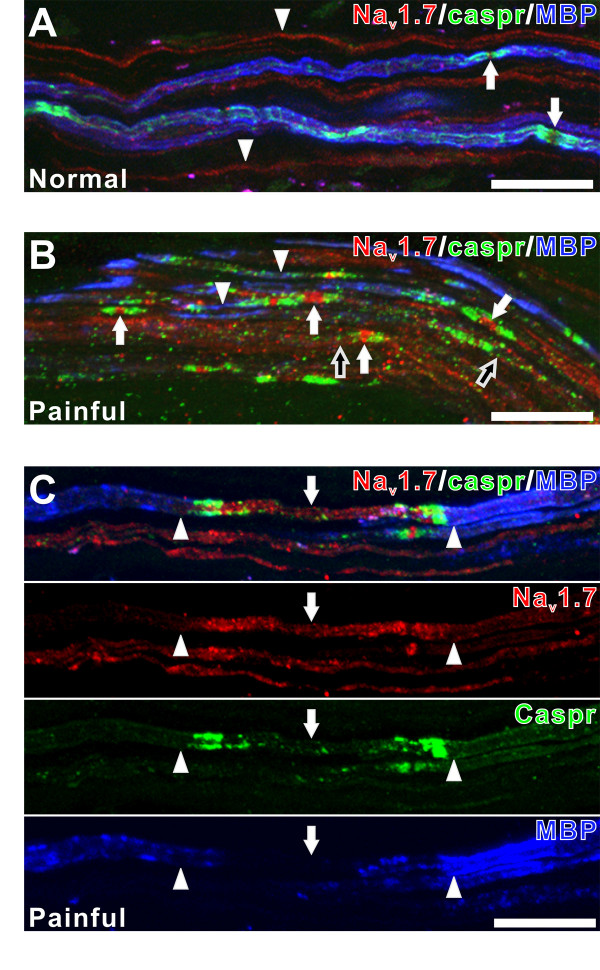
**Na_v_1.7 expression at caspr-identified nodal sites as related to state of myelination**. **A-C. **Confocal micrographs of collapsed z-projection images (five z-sections with spacing increments of one μm) showing Na_v_1.7 (red), caspr (green) and myelin basic protein (MBP; blue) staining relationships in one normal (A) and two painful (B and C) samples. **A **and **B. **The normal sample (A) shows the expression of MBP and caspr associated with some myelinated fibers (arrows), while this expression is absent in other smaller fibers that most likely lack myelin (arrowheads). The expression of Na_v_1.7 is prominent within the fibers that lack myelin. In contrast, the painful sample seen in (B) contains many fibers with prominent Na_v_1.7 expression at caspr-associated nodal sites (white arrows), but that show either alterations (arrowheads) or a lack of MBP staining altogether (black arrows). **C. **Combined and single channel images of Na_v_1.7, caspr, and MBP staining relationships seen in a painful sample showing the increased expression of Na_v_1.7 within an axon segment that is flanked on both sides with caspr and that lacks MBP (arrows), whereas Na_v_1.7 expression is less in the areas of this same axon where MBP expression is present (arrowheads). Scale bars = 20 μm.

Pulpal tissue sections that were triple-stained with Na_v_1.7, caspr and MBP antibodies allowed a characterization of Na_v_1.7 expression at typical and atypical nodal sites in relation to state of myelination as reflected with the use of the MBP antibody (Fig. 5). The expression of Na_v_1.7 in normal samples was seen evenly distributed within small fibers that lacked caspr and MBP staining and that most likely represent unmyelinated fibers, while expression within myelinated fibers with caspr and MBP stainings was mostly confined to the nodal site of some fibers (Fig. 5A). The expression of MBP within normal samples was prominent on the surface of fibers located within axon bundles and less at nodal regions (Fig. 5A). In contrast, this expression of MBP was altered in painful samples (Fig. 5B,C). Alterations in MBP included generalized (Fig. 5B) and focal (Fig. 5C) decreases in expressions. Generalized loss was seen in some fibers with typical nodes (Fig. 5B), while localized alterations included a loss of MBP in the area adjacent to heminodes (Fig. 5B) and the axonal region between split nodes (Fig. 5C). Typical and atypical nodes that were associated with a generalized loss of MBP staining (Fig. 5B) and the axonal area located between the split nodes (Fig. 5C), both contained prominent Na_v_1.7-immunofluorescence. These findings are consistent with a remodeling of NaChs in areas of demyelination as identified by a loss of MBP staining.

## Discussion

The results of this study demonstrate both the axonal expression of the Na_v_1.7 sodium channel (NaCh) isoform within the normal human dental pulp and changes in the expression of this isoform seen in painful samples. Quantitative methods showed an increased overall nerve fiber expression of Na_v_1.7 within axonal bundles but with no difference in expression within fibers located in the pulp horn, while single fiber analysis of caspr-identified nodal sites showed an increased expression of the isoform at typical and atypical nodal sites. Additionally, the atypical nodal sites were more common in painful samples and were associated with areas where MBP staining was lost. This pattern of MBP staining loss is similar to that found in demyelinating diseases [8] and this finding suggests the reorganization of NaChs at demyelinated sites as a pulpal pain mechanism. Together, these findings are consistent with a possible role for Na_v_1.7 in the constant, increased evoked and spontaneous pain sensations that characterize toothache and suggest the involvement of Na_v_1.7 as an ion channel involved with human inflammatory pain conditions. Furthermore, these changes are similar to those seen after inflammatory nerve lesions and suggest that pulpal pain mechanisms may include a neuroinflammatory neuropathic pain component.

The activation of voltage-gated NaChs is essential to action potential initiation and propagation and changes in expression may contribute to both inflammatory and neuropathic pain mechanisms [2]. Although evidence implicates the involvement of various NaCh isoforms in this process, recent findings in humans identify a critical role for the Na_v_1.7 isoform in pain sensation. These findings include mutations that result in nonfunctional channels and the inability to perceive acute painful stimuli [9,10], and point mutations that result in enhanced activation or impaired inactivation of channel current that are found in subjects with two chronic pain conditions that are characterized by burning-type pain [11,12].

The Na_v_1.7 isoform is prominently expressed within the dorsal root ganglia in humans and experimental animals [13,14] and especially in small diameter neurons that give rise to C-fibers and that respond to nociceptive stimuli [15]. Knockdown [16] and knockout [17] experiments also link the involvement of Na_v_1.7 to inflammatory hyperalgesia. Although these studies demonstrate a critical role for Na_v_1.7 in acute and inflammatory human pain, contributions to neuropathic pain mechanisms are less clear [18]. For example, the accumulation of Na_v_1.7 appeared greater in painful human neuromas when compared to nonpainful ones [19], yet a quantitative evaluation within human lingual nerve neuromas suggests that Na_v_1.7 expression alone is not correlated with the presence of painful dysesthesia [20]. Even though carious lesions of teeth result in pulpal pain that is most commonly thought to involve inflammatory mechanisms [21], certain aspects of pulpal pain may result from neuropathic pain processes that may contribute to the development of spontaneous pain that is present in some cases of toothache and that is a critical component of neuropathic pain [22]. Therefore, evaluations of Na_v_1.7 expression within both normal and painful pulpal samples may be useful to further our understanding regarding the role of Na_v_1.7 to both peripheral human inflammatory and neuropathic pain mechanisms.

The human dental pulp is richly innervated by nociceptors that include significant and differential contributions from both C-fibers and thinly myelinated fibers to discrete regions within the pulp [23,24]. The pain associated with irreversible pulpitis most likely involves contributions from both fiber types [25]. The pulpal expression of Na_v_1.7 was present within small fibers that lacked caspr (most likely representing C-fibers) and that are important in mediating dull toothache sensations, while the increased expression at nodal sites may be related to sharp, shooting spontaneous pain that was present in our patient population. Interestingly, many pulpal axons expressed N52. This result is surprising given that unmyelinated afferents are common within the pulp and others have shown little or no N52 expression in unmyelinated afferents [26]. The prominent expression of N52 within the pulp is even more intriguing given our finding that demonstrates Na_v_1.7 expression in presumptive unmyelinated fibers that lack markers (MBP and caspr) for myelinated fibers. One possible explanation for this finding includes an expression of Na_v_1.7 with N52 within the unmyelinated segments of myelinated afferents as they become free nerve endings. Furthermore, this broad expression of N52 suggests a possible thinning of fibers even before they enter the pulp and if so, this would overestimate the number of unmyelinated fibers reported within the pulp [5]. Additional studies are needed to further evaluate these staining relationships and the relative contributions of myelinated and unmyelinated fibers to overall pulpal innervation.

The presence of sharp, shooting spontaneous and especially lingering pain responses following sensory stimulation represents an important transition from reversible pulpitis to irreversible pulpitis and an important change from a hyperalgesic state to an acute pain condition. The changes seen within axon bundles in our painful sample population may be associated with this transition to an acute pain state. A noteworthy finding was that the painful samples showed an extensive small cell and most likely inflammatory cell infiltrate within coronal regions of the pulp that often surrounded intact fiber bundles, whereas smaller incipient lesions were not seen (data not shown). Since inclusion criteria for the painful group included the presence of spontaneous and moderate-to-severe pain, this finding suggests a correlation of extent of lesion with the transition to an acute pain state. This finding also suggests that pain severity due to pulpal disease involves either a cumulative effect on many fibers or a more selective effect on fibers deeper within the pulp, rather than the select activation of fibers located at the pulp periphery, such as those found within the odontoblastic layer of the pulp horn. It is possible that smaller lesions that are confined to the highly innervated and more superficial regions of the pulp such as the pulp horn may be more important in hyperalgesic pain responses and even reparative processes rather than the generation of acute and especially severe pulpal pain sensations. The overall pattern and extent of Na_v_1.7 expression seen in the painful human dental pulp not only supports contributions to this inflammatory pain condition, but also suggests the involvement of specific fibers types to the transition from a hyperalgesic to acute pulpitis pain state.

The increased incidence of atypical nodal forms seen in our painful samples and the association of Na_v_1.7 with these atypical nodal forms provides evidence for the remodeling of NaChs at demyelinated sites as a pulpal pain mechanism. Changes within myelinated fibers are important since the pain of irreversible pulpitis includes spontaneous and evoked pain responses characterized by a sharp, shooting quality that are most likely mediated by myelinated fibers. Many studies have demonstrated that a disruption of myelin produces profound changes in NaCh expression [27-30] and that heminodes and split nodes result from segmental and paranodal demyelination, respectively [31]. Areas of demyelination are characterized by a loss of myelin staining as identified with antibodies against MBP [8], myelin oligodendrocyte glycoprotein [32] and myelin-associated glycoprotein [31], with a pattern that is similar to that identified in the present study. Together these findings suggest that demyelinating influences exist in the inflamed dental pulp and that the reorganization of ion channels at these sites may contribute to activation of pulpal nociceptors. Although significant demyelination of axons can lead to conduction loss, demyelinating diseases that affect the peripheral and central nervous systems are often times associated with pain [33,34]. It is possible that conduction loss may represent an advanced disease stage, while more subtle and localized regions of demyelination could contribute to the activation of nociceptors and pain due to ion channel remodeling within these sites. Certainly the immune system response and the effect of this response on glia that provide important molecular signals that influence ion channel localization within axons, are all involved [35]. Further studies are needed to more fully understand the complexities of these immune-glial-neuronal interactions and their contributions to the development of an acute pain state.

The physiological implications related to the increased expression of Na_v_1.7 within the painful human dental pulp are unknown but most likely relate to increased neuronal excitability. Also unknown is the contribution of increased Na_v_1.7 expression at typical and atypical nodal sites to nerve activity and the pain experience. It is possible that the decreased expression of N52 seen in some fibers is associated with a degenerative response that leaves the fibers incapable of action potential propagation, but even so, more proximal portions of these fibers may remain physiologically active. One of the important findings in our study that may address this issue was the common occurrence of these forms in painful samples. These changes were not limited to areas adjacent to inflammatory lesions but rather were also seen at more distant sites (such as axon bundles in the radicular pulp). Neuronal changes seen within the diseased pulp must be critical to the pain experience since pulp removal or extraction of the tooth is typically highly effective in the rapid elimination of the pain. This finding suggests activation of nociceptors within diseased tissues is of clinical importance in acute pain conditions and strengthens the usefulness of single fiber analysis within peripheral tissues as possibly even more important than changes within other levels of the neuroaxis such as the neuronal cell body or central axon terminals. However, in some cases, endodontic therapy or tooth removal does not eliminate the pain and the pain becomes chronic [36] and in these cases changes at other levels are most certainly important.

Local anesthesia failures represent a major challenge to the practice of painless dentistry. Although these failures occur with the delivery of routine restorative procedures, the incidence increases dramatically when treating painful teeth with a diagnosis of irreversible pulpitis [37]. Since local anesthetics target NaChs, this increased incidence may include a change in NaCh expression. If a change in NaCh expression is responsible for this phenomenon, the change must be widespread along nerve fibers that innervate these teeth since local anesthesia failures are also seen where nerve blocks are performed at sites distant from the site of disease. Even so, local anesthesia failures may involve an increased density of NaChs that possibly includes an increase in isoforms that are more resistant to commonly used local anesthetics such as lidocaine. Although earlier reports suggested that the tetrodotoxin (TTX)-resistant NaCh isoforms may be less sensitive to local anesthetics [38], other evidence suggests the opposite, since TTX-resistant NaCh (including Na_v_1.8) currents appear to be more sensitive to lidocaine than TTX-sensitive forms such as Na_v_1.7 [39,40]. The increased expression of Na_v_1.7 within axon bundles of painful samples and the decreased effectiveness of lidocaine on TTX-sensitive NaCh isoforms, both suggest the possible involvement of Na_v_1.7 in local anesthesia failures. Although this possibility is appealing, a critical analysis of NaCh expression at more distant sites that correspond to areas where local anesthetics are typically applied would be necessary to more fully test this hypothesis.

Finally, the identification of atypical nodal forms within axons in the painful human tooth with an inflammatory lesion is similar to those seen in an experimental animal model for inflammatory neuropathic pain [41]. This provides evidence for neuroinflammatory influences in the genesis of toothache pain and furthermore suggests similar mechanisms may exist among certain chronic inflammatory and especially those neuropathic pain conditions that often have an inflammatory component [42]. It has even been suggested that a treatment modality for neuropathic pain following peripheral nerve injury may include the excision of the injured nerve, somewhat akin to pulp removal or extraction of the offending tooth [43]. In this regard, additional studies of the painful human dental pulp represent an important target to further our understanding of both inflammatory and neuropathic pain mechanisms.

## Conclusion

Our findings identify increased axonal expression of Na_v_1.7 within the painful human dental pulp that includes an increased expression at intact and remodeling/demyelinating nodal sites. These changes suggest contributions of Na_v_1.7 to the constant, increased evoked and spontaneous pain sensations that characterize toothache pain and to other acute and chronic inflammatory and neuropathic pain conditions.

## Methods

### Sample collection and tissue processing

This study was approved by the Human Subjects Institutional Review Board at the University of Texas Health Science Center at San Antonio and informed consent was obtained from all subjects who participated in the study. Teeth were obtained from subjects having an extraction of a normal, nonpainful third molar with fully formed apices (n = 13; 10 females and 3 males with an age range of 19–47), or a painful molar tooth diagnosed with irreversible pulpitis (n = 13; 10 females and 3 males with an age range of 24–49). Painful samples were limited to those that were associated with self-reports of moderate-to-severe levels of pain severity and the presence of spontaneous pain episodes as rated over the 24 hour time-period preceding the extraction. All painful teeth had the presence of a carious lesion that extended into the pulpal tissues, whereas those with large necrotic pulpal lesions were excluded.

Extracted teeth were collected in 0.1 M phosphate buffer (PB) and stored at 4°C. Later the same day, the teeth were split longitudinally and the pulpal tissues were removed and fixed in 4% paraformaldehyde in 0.1 M PB for 20 minutes. The pulpal tissue was rinsed in 0.1 M PB and then placed in 30% sucrose in 0.1 M PB overnight at 4°C. The next day the pulp was placed in Neg-50 (Richard-Allan Scientific; Kalamazoo, MI) and stored at -80°C. Pulpal samples were thawed and embedded in Neg-50, with a normal sample next to a painful sample, and serially sectioned with a cryostat at 30 μms in the longitudinal plane. Sections were placed onto Superfrost Plus slides (Fisher Scientific, Pittsburgh, PA), air dried and then stored at -20°C.

### Immunocytochemistry

Primary antibodies: rabbit polyclonal anti-Na_v_1.7 [13] (produced against a 15 amino acid sequence in the rat that shows 13/15 similarities with the human sequence, 1:100 for caspr analysis and 1:200 for nerve area analysis), mouse monoclonal anti-neurofilament 200 kD (N52, Sigma-Aldrich, St. Louis, MO, catalog #N0142, 1:2000), guinea-pig polyclonal anti-protein gene product 9.5 (PGP9.5, Chemicon, Temecula, CA, catalog # AB5898, 1:300), mouse monoclonal anti-caspr (developed by and/or obtained from the UC Davis/NINDS/NIMH Neuromab Facility, supported by NIH grant U24NS050606 and maintained by the Department of Pharmacology, School of Medicine, University of California, Davis, CA 95616, catalog #75-001, 1:500), rat monoclonal anti-myelin basic protein (MBP; Chemicon, Temecula, CA, catalog #MAB386 1:200). Secondary antibodies: Alexa Fluor^® ^568 goat anti-rabbit IgG, Alexa Fluor^® ^488 goat anti-mouse IgG, Alexa Fluor^® ^633 goat anti-rat IgG and goat anti-guinea-pig IgG (all from Molecular Probes, Eugene, OR). All secondary antibodies were used at 1:100.

Immunostaining was performed as described previously [44]. Briefly, tissue sections were permeabilized and blocked for non-specific protein binding sites with blocking solution consisting of 4% normal goat serum (Sigma), 2% bovine gamma-globulin (Sigma), and 0.3% Triton X-100 (Fisher Scientific) in PBS for 90 minutes prior to the incubation with primary antibodies in blocking solution for 16 hours. One section from each normal/painful pair was stained with Na_v_1.7/N52/PGP9.5, while the adjacent section from select sample pairs were stained with Na_v_1.7/caspr/MBP. Sections were rinsed with PBS, incubated in secondary antibody in blocking solution for 90 minutes, rinsed in PBS and ddH_2_O, dried, and coverslipped with Vectashield (Vector Laboratories, Burlingame, CA). All procedures were performed at room temperature.

### Confocal microscopic evaluation and image acquisition

Sections were evaluated with a Nikon Eclipse 90i C1si laser scanning confocal microscope with a 40×/1.30 N.A. oil immersion objective. This evaluation included the selection of a laser gain setting used to image Na_v_1.7-immunofluorescence to avoid saturated pixels so to allow a full dynamic range of Na_v_1.7-immunofluorescence pixel intensity and to select laser gain settings used to image all other primary antibodies. A series of optical images at 6 μm increments along the "z" axis of the sections stained with Na_v_1.7/N52/PGP9.5 antibodies were acquired from the middle 18 μm of each 30 μm thick section in the pulp horn and axon bundles in the coronal and radicular regions when present. Images obtained from the pulp horn in painful samples included only those areas where inflammatory cells were seen nearby to fibers. Care was taken to select fiber bundles that showed N52/PGP9.5 staining within intact fibers, since extensively fragmented fibers may represent degenerating ones. These selection criteria allowed the collection of a z-series of optical images from the pulp horn in 8 normal and 6 painful samples, coronal axon bundles in 12 normal and 8 painful samples, and radicular axon bundles in 8 normal and 8 painful samples. A z-series of optical images were also obtained at 1 μm increments from the middle 20 μm of the 30 μm thick sections of coronal and radicular axon bundles in four normal and four painful samples stained with Na_v_1.7/caspr/MBP antibodies. All images were captured with identical settings (including laser gain settings) at a 1024 × 1024 resolution (where each pixel is ≈ 0.3 μm width with an area ≈ 0.09 μm^2^) and saved as 12 bit single-channel images with a 0–4095 range of pixel intensities. Images were processed for illustration purposes by using Adobe Photoshop CS2 (Adobe Systems, San Jose, CA) and CorelDRAW 12 (Corel Corporation, Ottawa, Canada). Control tissue specimens were processed as above except the undiluted Na_v_1.7 antibody was preincubated with peptide antigen (approximately 30:1 peptide to antibody molar concentration ratio) for a minimum of 4 hours before application of the peptide-blocked antibody. Additional control specimens lacked the application of primary antibodies. Control sections lacked specific immunofluorescence.

### Quantitative and image analyses

The specimens stained with the Na_v_1.7/N52/PGP9.5 antibodies were used to evaluate Na_v_1.7 expression within N52 and PGP9.5 identified nerve fibers. Each individual channel was opened with NIH ImageJ software [45] with the use of the ICS Opener plug-in [46]. The corresponding PGP9.5 and N-52 z-slices were combined by using the "Max Operation" in the Image Calculator. The staining intensities in all images were filtered by applying a threshold value to remove low intensity pixels that represent nonspecific/background values. The threshold value was determined with mean and standard deviation pixel intensity values obtained from a histogram analysis of every Na_v_1.7 and combined N52/PGP9.5 image slice from normal samples. Average values for mean and standard deviation pixel intensities were then obtained independently for the Na_v_1.7 and combined N52/PGP9.5 groups, and then a threshold value (mean + 2 times the standard deviation) for each group was applied to remove (filter) background staining. This thresholding process was applied in a consistent manner to all images from the normal and painful sample groups and resulted in images in which N52/PGP9.5 staining was clearly limited to nerve fibers. The thresholded nerve fiber area was recorded by creating a selection in every slice and this was saved as a region of interest (ROI). The nerve area ROI was transposed onto the corresponding thresholded Na_v_1.7 image. The thresholded image was then redirected to the original Na_v_1.7 image so that when it was analyzed, the actual Na_v_1.7-immunofluorescence pixel intensities within the ROI were recorded. The particles were then analyzed, and the average intensity and percent area of nerve fiber occupied by Na_v_1.7 were recorded.

The Na_v_1.7 expression within single fibers at caspr-identified nodal sites that were classified as either typical or atypical were determined in the z-series of images that were obtained in sections stained with the Na_v_1.7 and caspr antibodies. The typical nodal sites were identified by extensive caspr staining within two bands that were separated by a nodal gap, while any deviations from this relationship were classified as atypical nodal sites. The most common caspr-identified atypical nodal site included heminodes that were identified as a single band of caspr staining, or two bands of caspr staining separated by a nodal gap but where the area of caspr staining was greatly diminished on one side to less than 50% of that seen on the side with more extensive caspr staining. All caspr-identified sites within single fibers were classified in each z-series image stack with navigation through each stack to clarify relationships, while the Na_v_1.7 expression at individual nodal sites was performed on maximum intensity collapsed z-projection images generated from each z-series. The Na_v_1.7-immunofluorescence pixel intensity in each collapsed image was filtered by applying a threshold value to remove low intensity pixels while leaving pixels with higher immunofluorescence intensity like those located within nodal accumulations. A threshold value for Na_v_1.7 staining was determined with mean and standard deviation pixel intensity values obtained from a histogram analysis of the single-channel Na_v_1.7-only maximum projection from each of the four normal tooth pulp samples. Mean values were calculated, and a threshold value (mean + six times the standard deviation) was applied to each maximum intensity collapsed z-projection image. All caspr-identified nodal sites were included in the analysis and Na_v_1.7-positive nodal sites were defined as those accumulations with one or more Na_v_1.7-positive pixel(s) above threshold that were clearly associated with caspr staining as confirmed with identification of the same nodal site in the appropriate z-series image stack. This analysis was limited to caspr-identified sites located within fibers that were fully represented within the z-series image stack. All nodal accumulations were classified as either typical, or atypical and further classified as associated with Na_v_1.7 or as lacking this association. This evaluation further allowed the characterization of Na_v_1.7 at some caspr-identified atypical sites that were classified as split nodes where two distinct Na_v_1.7 accumulations were separated by a gap in the Na_v_1.7 staining within the same fiber and with each Na_v_1.7 accumulation being flanked on only one side by caspr.

### Statistical analysis

Statistical analysis to determine significance used the unpaired Student *t*-test while error bars on all graphs represent the standard error of the mean (SEM). The "n" values used in all instances represented the number of samples evaluated.

## List of abbreviations

MBP: myelin basic protein; ROI: region of interest; SEM: standard error of the mean; NaCh(s): sodium channel(s); N52: anti-Neurofilament 200 kD; TTX: tetrodotoxin. 

## Competing interests

The authors declare that they have no competing interests.

## Authors' contributions

SL assisted with tissue preparation and staining, collection of data, data analysis, and helped draft the manuscript. GP assisted with tissue staining, collection of data, and data analysis. SRL helped to conceive the study, participated in its design, produced the Na_v_1.7 antibodies, guided data analysis, and helped draft the manuscript. MH conceived the study, participated in its design and coordination, assisted with tissue preparation, and drafted the manuscript. All authors read and approved the final manuscript.

## References

[B1] Hille B (2001). Ion Channels of Excitable Membranes.

[B2] Amir R, Argoff CE, Bennett GJ, Cummins TR, Durieux ME, Gerner P, Gold MS, Porreca F, Strichartz GR (2006). The role of sodium channels in chronic inflammatory and neuropathic pain. J Pain.

[B3] Goldin AL, Barchi RL, Caldwell JH, Hofmann F, Howe JR, Hunter JC, Kallen RG, Mandel G, Meisler MH, Netter YB, Noda M, Tamkun MM, Waxman SG, Wood JN, Catterall WA (2000). Nomenclature of voltage-gated sodium channels. Neuron.

[B4] Cummins TR, Sheets PL, Waxman SG (2007). The roles of sodium channels in nociception: Implications for mechanisms of pain. Pain.

[B5] Nair PN (1995). Neural elements in dental pulp and dentin. Oral Surg Oral Med Oral Pathol Oral Radiol Endod.

[B6] Lipton JA, Ship JA, Larach-Robinson D (1993). Estimated prevalence and distribution of reported orofacial pain in the United States. J Am Dent Assoc.

[B7] Poliak S, Gollan L, Martinez R, Custer A, Einheber S, Salzer JL, Trimmer JS, Shrager P, Peles E (1999). Caspr2, a new member of the neurexin superfamily, is localized at the juxtaparanodes of myelinated axons and associates with K+ channels. Neuron.

[B8] Trapp BD, Peterson J, Ransohoff RM, Rudick R, Mork S, Bo L (1998). Axonal transection in the lesions of multiple sclerosis. N Engl J Med.

[B9] Cox JJ, Reimann F, Nicholas AK, Thornton G, Roberts E, Springell K, Karbani G, Jafri H, Mannan J, Raashid Y, Al-Gazali L, Hamamy H, Valente EM, Gorman S, Williams R, McHale DP, Wood JN, Gribble FM, Woods CG (2006). An SCN9A channelopathy causes congenital inability to experience pain. Nature.

[B10] Goldberg YP, MacFarlane J, MacDonald ML, Thompson J, Dube MP, Mattice M, Fraser R, Young C, Hossain S, Pape T, Payne B, Radomski C, Donaldson G, Ives E, Cox J, Younghusband HB, Green R, Duff A, Boltshauser E, Grinspan GA, Dimon JH, Sibley BG, Andria G, Toscano E, Kerdraon J, Bowsher D, Pimstone SN, Samuels ME, Sherrington R, Hayden MR (2007). Loss-of-function mutations in the Nav1.7 gene underlie congenital indifference to pain in multiple human populations. Clin Genet.

[B11] Yang Y, Wang Y, Li S, Xu Z, Li H, Ma L, Fan J, Bu D, Liu B, Fan Z, Wu G, Jin J, Ding B, Zhu X, Shen Y (2004). Mutations in SCN9A, encoding a sodium channel alpha subunit, in patients with primary erythermalgia. J Med Genet.

[B12] Fertleman CR, Baker MD, Parker KA, Moffatt S, Elmslie FV, Abrahamsen B, Ostman J, Klugbauer N, Wood JN, Gardiner RM, Rees M (2006). SCN9A mutations in paroxysmal extreme pain disorder: allelic variants underlie distinct channel defects and phenotypes. Neuron.

[B13] Toledo-Aral JJ, Moss BL, He ZJ, Koszowski AG, Whisenand T, Levinson SR, Wolf JJ, Silos-Santiago I, Halegoua S, Mandel G (1997). Identification of PN1, a predominant voltage-dependent sodium channel expressed principally in peripheral neurons. Proc Natl Acad Sci U S A.

[B14] Sangameswaran L, Fish LM, Koch BD, Rabert DK, Delgado SG, Ilnicka M, Jakeman LB, Novakovic S, Wong K, Sze P, Tzoumaka E, Stewart GR, Herman RC, Chan H, Eglen RM, Hunter JC (1997). A novel tetrodotoxin-sensitive, voltage-gated sodium channel expressed in rat and human dorsal root ganglia. J Biol Chem.

[B15] Djouhri L, Newton R, Levinson SR, Berry CM, Carruthers B, Lawson SN (2003). Sensory and electrophysiological properties of guinea-pig sensory neurones expressing Nav 1.7 (PN1) Na+ channel alpha subunit protein. J Physiol.

[B16] Yeomans DC, Levinson SR, Peters MC, Koszowski AG, Tzabazis AZ, Gilly WF, Wilson SP (2005). Decrease in inflammatory hyperalgesia by herpes vector-mediated knockdown of Nav1.7 sodium channels in primary afferents. Hum Gene Ther.

[B17] Nassar MA, Stirling LC, Forlani G, Baker MD, Matthews EA, Dickenson AH, Wood JN (2004). Nociceptor-specific gene deletion reveals a major role for Nav1.7 (PN1) in acute and inflammatory pain. Proc Natl Acad Sci U S A.

[B18] Nassar MA, Levato A, Stirling LC, Wood JN (2005). Neuropathic pain develops normally in mice lacking both Nav1.7 and Nav1.8. Mol Pain.

[B19] Kretschmer T, Happel LT, England JD, Nguyen DH, Tiel RL, Beuerman RW, Kline DG (2002). Accumulation of PN1 and PN3 sodium channels in painful human neuroma-evidence from immunocytochemistry. Acta Neurochir (Wien).

[B20] Bird EV, Robinson PP, Boissonade FM (2007). Na(v)1.7 sodium channel expression in human lingual nerve neuromas. Arch Oral Biol.

[B21] Byers MR, Narhi MV (1999). Dental injury models: experimental tools for understanding neuroinflammatory interactions and polymodal nociceptor functions. Crit Rev Oral Biol Med.

[B22] Thomas PK (1984). Clinical features and differential diagnosis of peripheral neuropathy. In Peripheral Neuropathy (Vol 2).

[B23] Pashley DH, Liewehr FR, Cohen S, Hargreaves KM (2006). Structure and functions of the dentin-pulp complex. Pathways of the Pulp.

[B24] Anderson DJ, Hannam AG, Mathews B (1970). Sensory mechanisms in mammalian teeth and their supporting structures. Physiol Rev.

[B25] Bender IB (2000). Pulpal pain diagnosis--a review. J Endod.

[B26] Lawson SN, Waddell PJ (1991). Soma neurofilament immunoreactivity is related to cell size and fibre conduction velocity in rat primary sensory neurons. J Physiol.

[B27] England JD, Gamboni F, Levinson SR (1991). Increased numbers of sodium channels form along demyelinated axons. Brain Res.

[B28] England JD, Gamboni F, Levinson SR, Finger TE (1990). Changed distribution of sodium channels along demyelinated axons. Proc Natl Acad Sci U S A.

[B29] Dugandzija-Novakovic S, Koszowski AG, Levinson SR, Shrager P (1995). Clustering of Na+ channels and node of Ranvier formation in remyelinating axons. Journal of Neuroscience.

[B30] England JD, Happel LT, Kline DG, Gamboni F, Thouron CL, Liu ZP, Levinson SR (1996). Sodium channel accumulation in humans with painful neuromas. Neurology.

[B31] Arroyo EJ, Sirkowski EE, Chitale R, Scherer SS (2004). Acute demyelination disrupts the molecular organization of peripheral nervous system nodes. J Comp Neurol.

[B32] Howell OW, Palser A, Polito A, Melrose S, Zonta B, Scheiermann C, Vora AJ, Brophy PJ, Reynolds R (2006). Disruption of neurofascin localization reveals early changes preceding demyelination and remyelination in multiple sclerosis. Brain.

[B33] Hadjimichael O, Kerns RD, Rizzo MA, Cutter G, Vollmer T (2007). Persistent pain and uncomfortable sensations in persons with multiple sclerosis. Pain.

[B34] Ehde DM, Osborne TL, Hanley MA, Jensen MP, Kraft GH (2006). The scope and nature of pain in persons with multiple sclerosis. Mult Scler.

[B35] Poliak S, Peles E (2003). The local differentiation of myelinated axons at nodes of Ranvier. Nat Rev Neurosci.

[B36] Polycarpou N, Ng YL, Canavan D, Moles DR, Gulabivala K (2005). Prevalence of persistent pain after endodontic treatment and factors affecting its occurrence in cases with complete radiographic healing. Int Endod J.

[B37] Hargreaves KM, Keiser K (2002). Local anesthetic failure in endodontics:. Mechanisms and Management. Endodontic Topics.

[B38] Scholz A, Kuboyama N, Hempelmann G, Vogel W (1998). Complex blockade of TTX-resistant Na+ currents by lidocaine and bupivacaine reduce firing frequency in DRG neurons. J Neurophysiol.

[B39] Chevrier P, Vijayaragavan K, Chahine M (2004). Differential modulation of Nav1.7 and Nav1.8 peripheral nerve sodium channels by the local anesthetic lidocaine. Br J Pharmacol.

[B40] Leffler A, Reiprich A, Mohapatra DP, Nau C (2007). Use-dependent block by lidocaine but not amitriptyline is more pronounced in tetrodotoxin (TTX)-Resistant Nav1.8 than in TTX-sensitive Na+ channels. J Pharmacol Exp Ther.

[B41] Henry MA, Freking AR, Johnson LR, Levinson SR (2006). Increased sodium channel immunofluorescence at myelinated and demyelinated sites following an inflammatory and partial axotomy lesion of the rat infraorbital nerve. Pain.

[B42] Said G, Hontebeyrie-Joskowicz M (1992). Nerve lesions induced by macrophage activation. Res Immunol.

[B43] Watson CP, Stinson JN, Dostrovsky JO, Hawkins C, Rutka J, Forrest C (2007). Nerve resection and re-location may relieve causalgia: a case report. Pain.

[B44] Alvarado LT, Perry GM, Hargreaves KM, Henry MA (2007). TRPM8 Axonal expression is decreased in painful human teeth with irreversible pulpitis and cold hyperalgesia. J Endod.

[B45] Rasband WS ImageJ. http://rsb.info.nih.gov/ij/.

[B46] Stuurman N ICS Opener plug-in. http://valelab.ucsf.edu/~nico/IJplugins/Ics_Opener.html.

